# Thioreductase-Containing Epitopes Inhibit the Development of Type 1 Diabetes in the NOD Mouse Model

**DOI:** 10.3389/fimmu.2016.00067

**Published:** 2016-03-02

**Authors:** Elin Malek Abrahimians, Luc Vander Elst, Vincent A. Carlier, Jean-Marie Saint-Remy

**Affiliations:** ^1^Center for Molecular and Vascular Biology, University of Leuven, Leuven, Belgium; ^2^ImCyse SA, Leuven, Belgium

**Keywords:** type 1 diabetes, NOD mouse, cytolytic CD4^+^ T cells, antigen-specific, MHC class II epitopes

## Abstract

Autoreactive CD4^+^ T cells recognizing islet-derived antigens play a primary role in type 1 diabetes. Specific suppression of such cells therefore represents a strategic target for the cure of the disease. We have developed a methodology by which CD4^+^ T cells acquire apoptosis-inducing properties on antigen-presenting cells after cognate recognition of natural sequence epitopes. We describe here that inclusion of a thiol-disulfide oxidoreductase (thioreductase) motif within the flanking residues of a single MHC class II-restricted GAD65 epitope induces GAD65-specific cytolytic CD4^+^ T cells (cCD4^+^ T). The latter, obtained either *in vitro* or by active immunization, acquire an effector memory phenotype and lyse APCs by a Fas–FasL interaction. Furthermore, cCD4^+^ T cells eliminate by apoptosis activated bystander CD4^+^ T cells recognizing alternative epitopes processed by the same APC. Active immunization with a GAD65 class II-restricted thioreductase-containing T cell epitope protects mice from diabetes and abrogates insulitis. Passive transfer of *in vitro*-elicited cCD4^+^ T cells establishes that such cells are efficient in suppressing autoimmunity. These findings provide strong evidence for a new vaccination strategy to prevent type 1 diabetes.

## Introduction

Although the initial event leading to type 1 diabetes remains elusive, many data converge, both in mice and in humans, to establish that T cell-mediated autoimmunity toward several pancreatic islet cell autoantigens plays a significant role in pathogenesis (1, 2). Activation of CD4^+^ T cells by presentation of β-cell antigens by APCs in the pancreas (3) and the regional draining lymph nodes (4) represents a first step in the initiation of autoimmunity, leading to organ inflammation and ultimately β-cell destruction. A therapy targeting the initial step in antigen recognition would therefore carry the potential of significantly impacting disease progression, provided such control remains antigen specific (5). Antigen-specific therapy offers the superiority of being limited only to responses specific to antigens involved in the disease process without exposing the body to a status of lessened reactivity to other antigenic challenges. Indeed, general immune suppression can lead to increased risk of infection and tumor proliferation and requires lifelong treatment.

Numerous studies have demonstrated the efficacy of antigen-based therapies, including GAD65 protein, peptides, or modified peptides, in preventing type 1 diabetes in non-obese diabetes (NOD) mice (6). These strategies are based either on a shift within CD4^+^ cells from a Th1 to a Th2 profile or on the induction of tolerance by the activation of regulatory T cells (Tregs). Previous attempts in this direction have failed in clinical trials and no disease-modifying agent has reached the market so far. There is therefore a need for developing a new immune-modulating strategy.

CD4^+^ T cells can be polarized into a cytolytic phenotype, thereby acquiring the capacity to induce apoptosis in APCs after peptide–MHC class II cognate recognition. This can be achieved only after synapse formation and by increasing the strength of the synapse created with peptide–MHC complexes obtained by adding a thioreductase motif within the flanking residues of class II-restricted epitopes (7). Importantly, APC apoptosis prevents activation of CD4^+^ T cells to alternative epitopes of the autoantigen from which the peptide is derived or to epitopes of associated autoantigens. An immune response to multiple antigens can therefore be prevented or suppressed. In addition, CD4^+^ T cells polarized into a cytolytic phenotype eliminate by apoptosis bystander CD4^+^ T cells provided they are activated at the surface of the same APC (8).

GAD65 is one of several β-cell antigens recognized by T cells and its importance in the pathogenesis of autoimmune diabetes has been repeatedly demonstrated (9, 10). Epitope selection was based on the previously described GAD65_524–538_ epitope as eliciting a CD4^+^ T cell response (11) and peptide-binding predictor algorithms (e.g., Rankpep). GAD65_528–538_ was selected based on both its description in the literature and as a good MHC class II binder. We therefore used a synthetic peptide encompassing this class II-restricted GAD65 epitope to determine whether insertion of a thioreductase motif within flanking residues could prevent type 1 diabetes. We further evaluated whether this effect could be related to the induction of cytolytic CD4^+^ T cells. Experiments were carried out in the most commonly used spontaneous model of the disease, the NOD mouse.

## Materials and Methods

### Mice

Female NOD/MrkTac mice were purchased from Taconic Europe (Laven, Denmark). Female *proinsulin* 2-deficient (*Ins2*^−/−^) NOD mice, generated by backcrossing *proinsulin* 2-deficient 129 mice onto the NOD background, were graciously provided by Boitard (INSERM, Institut Cochin, Paris, France). All animal care and experiments were conducted according to the Institutional Animal Care and Research Advisory Committee of the University of Leuven. According to the requirements of the experiment, mice were housed either in conventional animal facilities or in specific pathogen-free (SPF) conditions at the University of Leuven.

### Peptides

Synthetic peptides encompassing a GAD65 class II-restricted epitope (e.g., GAD65_528–538_, KVAPVIKARMM reported as WTGAD65), the same epitope containing a thioreductase motif of the CxxC format, wherein C stands for cysteine and x for any amino acid in flanking residues (reported as CCGAD65), the loss-of-function sequence where the CxxC motif is replaced by a AxxA motif, wherein A stands for alanine (reported as AAGAD65), a non-relevant hen egg lysozyme (HEL) class II-restricted epitope (NTDGSTDYGILQINSR reported as WTHEL), and the CxxC-containing counterpart (reported as CCHEL) were produced by solid phase Fmoc chemistry (Eurogentec, Liège, Belgium). Purity of ≥95% was verified by chromatography.

### Peptide Immunization and Diabetes Monitoring

Four-week-old female NOD mice were immunized with designated peptide in alum (Alum Imject^®^, Pierce, Rockford, IL, USA), using 50 μg of peptide, for four subcutaneous weekly injections. Diabetes development was monitored until 40 weeks of age by weekly blood glucose measurements with One Touch Vita^®^ glucometer (LifeScan, Johnson & Johnson Company, Milpitas, CA, USA), and diabetes was diagnosed when blood glucose levels were ≥300 mg/dl on two consecutive measurements (12).

### Induction and Culture of Antigen-Specific CD4^+^ T Cells

Female NOD mice were immunized by four subcutaneous injections of 50 μg peptide in alum at 1-week intervals; spleen CD4^+^ T cells were isolated 2 weeks after the last injection (CD4 T cell isolation kit, Miltenyi, Bergisch Gladbach, Germany). T cell-depleted splenocytes (reported as APCs) (CD90.2 microbeads, Miltenyi) from naive female NOD mice were preloaded for 2 h with peptide (5 μM) and treated with Mitomycin-C^®^ (Kyowa, Tokyo, Japan). CD4^+^ T cells were stimulated with APCs loaded with peptide in a T to APC ratio of 1:3 and cultured in enriched RPMI 1640 medium containing 5% FCS, 50 μM 2-ME and 200 μg/ml Gentamicin (Invitrogen Life Technologies, Gent, Belgium). After 10–14 days, cells were restimulated in the same conditions but with 10 U/ml mouse IL-2 (Roche, Brussels, Belgium). All cells described as cCD4^+^ T cells were generated identically by isolating CD4^+^ T cells from CCGAD65-immunized mice and expanding them *in vitro* in the presence of CCGAD65-loaded APCs.

### Cell Proliferation

CD4^+^ T cells were cultured for 4 days with Mitomycin-C^®^-treated T cell-depleted splenocytes with the indicated amount of peptides. ^3^H-thymidine (1 μCi/well, PerkinElmer, Zaventem, Belgium) was added for the last 18 h before scintillation counting.

### Cell Staining

Fluorochrome-stained antibodies recognizing mouse CD3e (145-2C11), CD4 (GK1.5), CD8 (SK1), CD25 (PC61), CD27 (LG.3A10), CD28 (37.51), CD44 (IM7), CD62-L (MEL-14), CD127 (SB/199), CD107a (1D4B), and GATA-3 (L50-823) were purchased from BD Biosciences (Erembodegem, Belgium). Fluorochrome-stained antibodies recognizing mouse Foxp3 (FJK-16) and T-bet (4B10) were from eBioscience (Frankfurt, Germany). Intracellular staining for T-bet, GATA-3, and Foxp3 was done with Foxp3 Staining Kit (eBioscience). All stainings were performed following manufacturers’ instructions. Samples were acquired on a FacsCantoII flow cytometer (BD Biosciences), and data were analyzed with FACSDiva™ software (BD Biosciences) and Weasel software (WEH Institute, Melbourne, VIC, Australia).

### Killing Assay

Spleen B cells were isolated (B cell isolation kit, Miltenyi) and cultured overnight in the presence of 25 μg/ml LPS (*Escherichia coli* 055:B5, Sigma-Aldrich, Diegem, Belgium) to maintain sufficient cell survival and support antigen presentation. Dead cells were removed by Ficoll centrifugation (Lympholyte-M, Cedarlane Labs, Atlanta, GA, USA) and remaining B cells were stained with Cyto-ID Red long-term cell tracer kit (Enzo Life Sciences, Lausen, Switzerland) following manufacturers’ instructions. B cells were then cocultured for 18 h with CD4^+^ T cells (ratio B:T, 1:5) in the presence of indicated peptide (2 μM, added to the culture media). Annexin V APC was used to detect cell death in B cells (Annexin V detection kit, BD Biosciences) according to manufacturers’ instructions. Gated B cells were then analyzed for Annexin V binding on flow cytometer. For inhibition of granzyme-B (GZB) activity, Z-AAD-CMK (Calbiochem/Merck, Overijse, Belgium) was added at 20 μg/ml during the entire coculture period. Inhibition of FasL was performed with functional grade anti-mouse CD178 antibody (clone MFL3, eBioscience) at 20 μg/ml during the coculture period.

### Bystander Suppression Assays

Target CD4^+^ T cells were labeled with 125 nM CFSE (Molecular Probes Life Technologies, Gent, Belgium) for 8 min in PBS at 37°C. The reaction was stopped by washing the cells with PBS-containing 2% FBS. These labeled cells were cocultured for 72 h with cytolytic CD4^+^ T cells and splenic B cells used as APCs (ratio target cells:cytolytic cells:APCs, 1:2:4) in the presence of the indicated peptides (5 μM, added to the culture media). Apoptosis was measured with Annexin V staining as described for the APC killing assay.

### Adoptive Transfer of CD4^+^ T Cells

Eight-week-old female NOD mice were immunized as described above with either AAGAD65 or CCGAD65. Two weeks after last injection, mice were sacrificed, and CD4^+^ T cells were isolated from spleen and expanded *in vitro*. Cultured cells (2.10^5^ cells/mouse) were transferred by intravenous injection to 6-week-old naive female NOD mice.

### Histology

Pancreases were fixed and embedded in paraffin, and 5–6 μm sections were stained with H&E to assess inflammatory infiltrates of the islets. Pancreatic islets were scored for the extent of insulitis, as previously described: grade 0, no inflammation; grade 1, peri-insulitis with inflammatory cells touching islet perimeters, but not penetrating; grade 2, inflammatory infiltration <25% of islet area; grade 3, inflammation >25% but <75% of islet area; and grade 4, inflammation >75% of islet area (end-stage insulitis). Insulitis index (II) was calculated as II = (0 × *n*0) + (1 × *n*1) + (2 × *n*2) + (3 × *n*3) + (4 × *n*4)/4 × (*n*0 + *n*1 + *n*2 + *n*3 + *n*4) (12). A minimum of 40 non-overlapping islets were observed per mouse. Images were acquired using Zeiss Axiovision 4.6 software on Leica DM RBE microscope equipped with a Zeiss AxioCam MRc5 digital camera.

### Statistical Analysis

Data are expressed as mean and SD or SEM as specified. Statistical evaluation was carried out with Prism 6.0. Differences in disease incidence were assessed by Mantel–Cox log-rank test analysis. Non-parametric assays were used for evaluating differences between means (Mann–Whitney test and ANOVA for multiple comparison tests). Significance was considered at *P* < 0.05.

## Results

### Immunization with CCGAD65 Inhibits the Development of Diabetes and Abrogates Insulitis in NOD Mice

Four-week-old female NOD mice were immunized with a peptide encompassing a class II-restricted GAD65 epitope and a thioreductase motif (CCGAD65) in conventional animal facilities. Glycemia was followed on a weekly basis until the age of 40 weeks. Diabetes-free survival rate was significantly increased for immunized (82%) versus untreated controls (38%) at 40 weeks of age (Figure S1A in Supplementary Material).

Leukocytic infiltration of the pancreatic islets (insulitis) is the major histopathological feature of type 1 diabetes development (12). Figure S1B in Supplementary Material shows significantly reduced insulitis index (II) at 40 weeks in normoglycemic CCGAD65-immunized mice with a mean II of 0.10 ± 0.03 (SEM) as compared to 0.45 ± 0.06 (SEM) for the untreated group. Notably, the immunized group benefits from a significantly higher number of insulitis-free islets with 75 ± 6% (SEM) intact islets as opposed to 34 ± 7% (SEM) in the untreated group, and 4 out of 16 mice in the treated group show absolutely no signs of insulitis at 40 weeks of age (Figures S1B,C in Supplementary Material).

**Figure 1:**
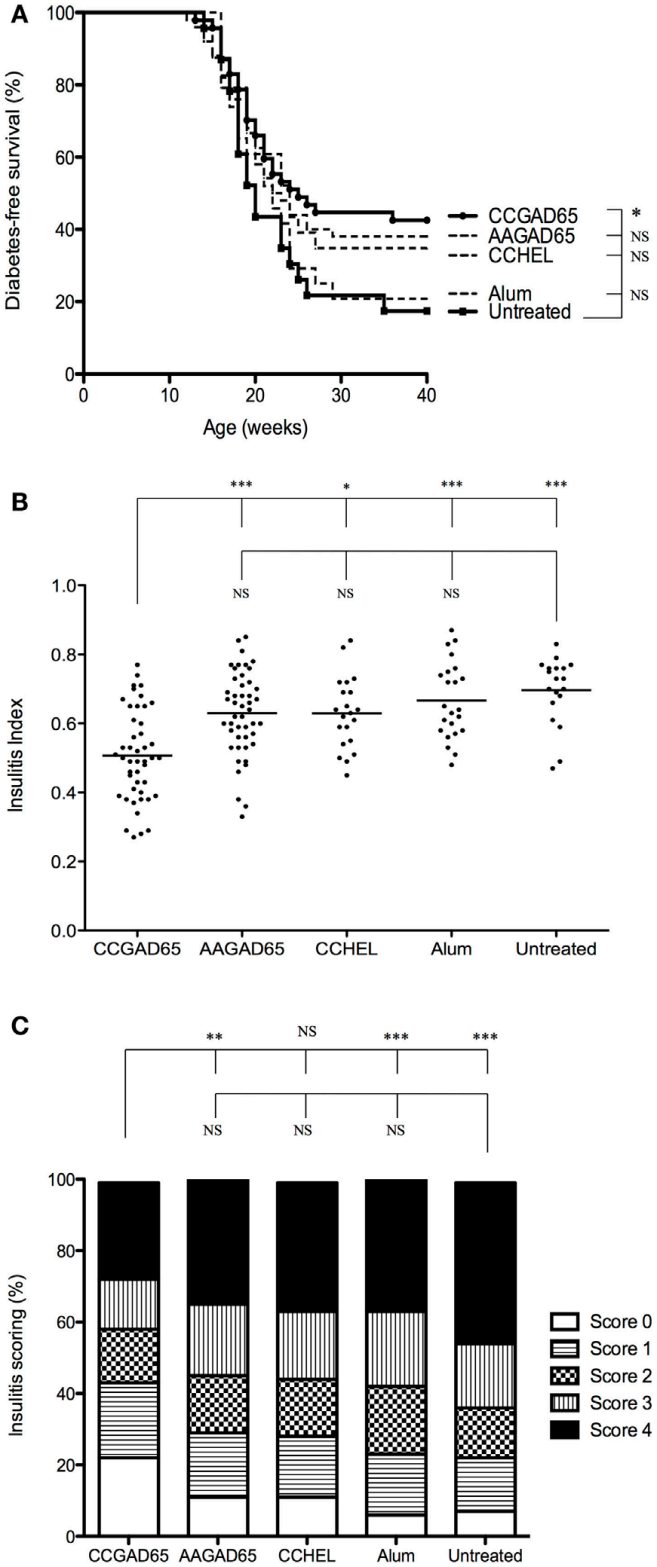
**NOD mice immunized with CCGAD65 peptide in SPF animal facilities**. **(A)** Four-week-old female NOD mice received four subcutaneous injections of 50 μg of peptide in alum with a 7-day interval. Mice were randomly divided in five cohorts of five groups each treated with the same immunization protocol. The first group was immunized with the CCGAD65 peptide in alum, the second with the loss-of-function AAGAD65 peptide in alum, the third group with the non-relevant CCHEL peptide in alum, the fourth group received alum alone, and the fifth group remained untreated. Results represent diabetes-free survival rates at, from top to bottom at 40 weeks, 43% for CCGAD65-immunized mice (*n* = 47), 38% for AAGAD65-immunized mice (*n* = 50), 35% for CCHEL-immunized mice (*n* = 23), 21% for alum-treated mice (*n* = 24), 17% for untreated control group (*n* = 23), *P < 0.05 and *P* > 0.1 reported as NS (Mantel–Cox log-rank test). **(B,C)** Pancreatic sections of mice sacrificed after two consecutive weekly glycemia above 300 mg/dl or after remaining normoglycemic during 40 weeks were stained with H&E and insulitis was scored by examining a minimum of 40 islets per mouse. **(B)** Individual II are represented for each group with line representing mean index; from left to right, CCGAD65 peptide in alum (*n* = 46, mean II = 0.51), AAGAD65 peptide in alum (*n* = 47, mean II = 0.63), CCHEL peptide in alum (*n* = 21, mean II = 0.63), alum alone (*n* = 23, mean II = 0.67), and untreated (*n* = 19, mean II = 0.70). Statistical significance was calculated with Kruskal–Wallis ANOVA test, *P* < 0.0001 followed by Dunn’s multiple comparison test as noted, *P < 0.05. **(C)** Grading of insulitis of the various groups as indicated. Statistical significance was calculated based on insulitis-free islets with Kruskal–Wallis ANOVA test, *P* < 0.0001 and Dunn’s multiple comparison test as indicated, *P < 0.05.

Diabetes onset in NOD mice can be affected by the microbial environment in the housing facility partly due to the interaction of the intestinal microbiota with the innate immune system (13, 14). To ensure efficacy of our setting, this preliminary experiment was repeated with mice kept under SPF facilities (15). Control mice further included four distinct settings. The first group was immunized with a peptide in which the thioreductase activity was abolished by substitution of the two cysteines of the motif by alanines and referred to as loss-of-function peptide. The second group was immunized with a non-relevant CCHEL peptide. The third group received the adjuvant alone and the last group remained untreated. Only CCGAD65-immunized group showed a diabetes-free survival curve significantly different from untreated group. Indeed, 43% of mice receiving the CCGAD65 peptide remained diabetes-free as opposed to 17% in untreated mice. All other groups were not significantly different from the untreated group (Figure 1A). Although diabetes incidence in CCGAD65-immunized was not statistically distinct from the other control groups, we systematically and repeatedly noted a lower incidence of diabetes in this group in comparison to the four other control groups. Histological assessment of inflammatory infiltrate further confirmed this trend.

Similar to others, we observed a consistent difference for diabetes incidence and severity of insulitis infiltration between NOD females housed in conventional or SPF animal facilities (15). Thus, the overall IIs were much higher for mice maintained in SPF conditions. Importantly, however, the calculated indexes of the CCGAD65 group remained significantly lower (Figure 1B) and with a better preservation of insulitis-free islets as compared to all the control groups (Figure 1C) even under such conditions. Note the highest percentage of islets without insulitis at 22% in CCGAD65 group, as opposed to 11% in AAGAD54 group, 11% in CCHEL group, 6% in alum group, and 8% in untreated group. Separating data in Figures 1B,C according to diabetes status do not modify the statistical superiority of CCGAD65 in diminishing insulitis. Furthermore, this reduction in insulitis can been seen at earlier stages as well as in 40-week-old mice indicating a sustainable effect (Figure S2 in Supplementary Material).

Overall, these data strongly suggest that active immunization with a thioreductase-containing GAD65 epitope inhibits the development of type 1 diabetes in female NOD mice both in less restrained conventional and in SPF conditions and confirms that mice are indeed protected from the autoimmune attack at the level of the islet. In addition, it is demonstrated that treatment neither with the corresponding loss-of-function peptide, nor with the non-relevant thioreductase-containing peptide, nor with the adjuvant alone can reproduce such protection.

### CCGAD65 Peptide Elicits CD4^+^ T Cells with a Unique Phenotype and Cytolytic Properties

**Figure 2:**
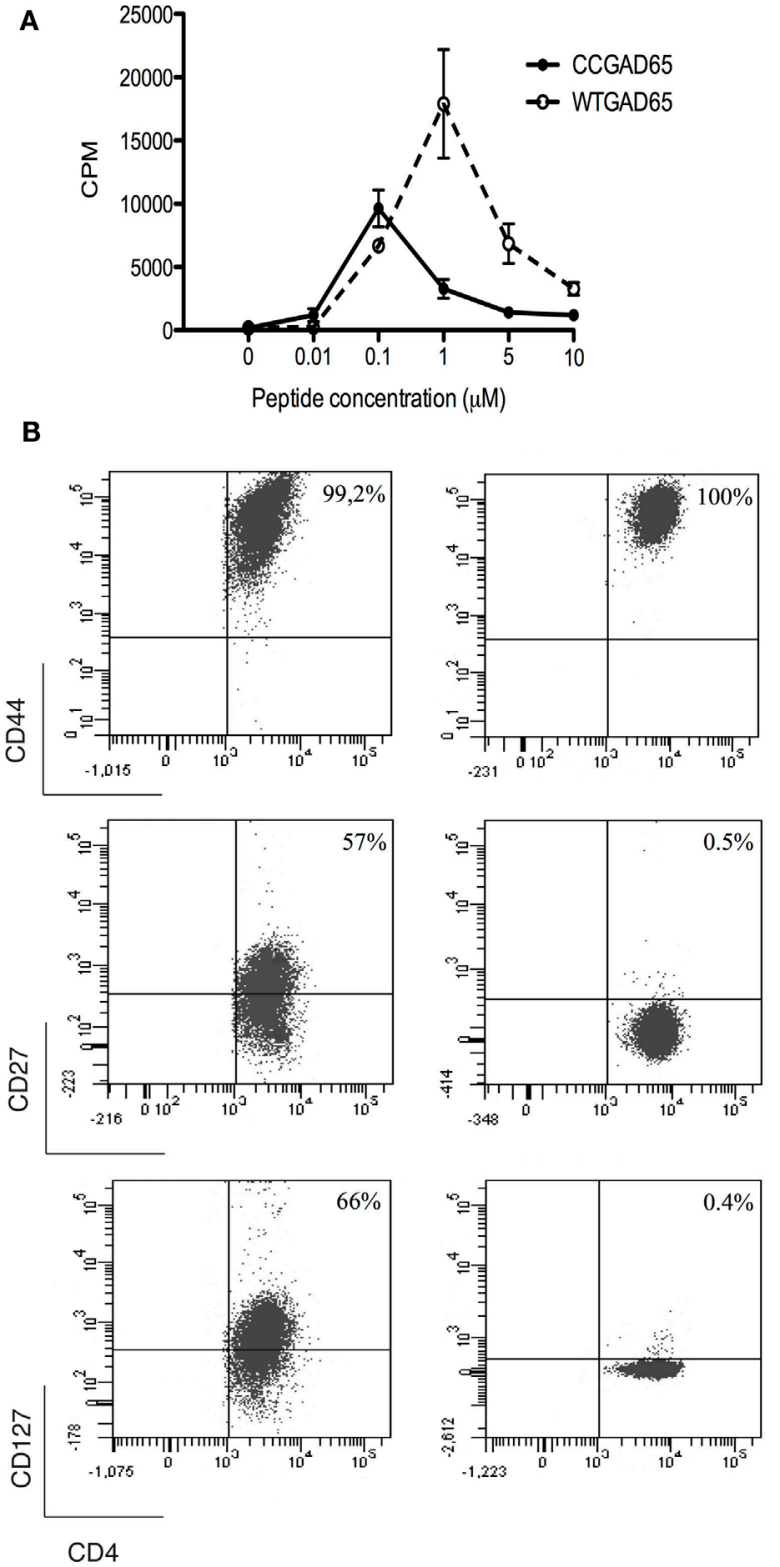
**Characterization of CCGAD65-induced CD4^+^ T cells**. **(A)** The proliferative response of CCGAD65-induced CD4^+^ T cells (pooled from three previously CCGAD65-immunized mice) to either CCGAD65 peptide (continuous line) or the natural counterpart (WTGAD65, dashed line) was assayed after 72 h in cocultures with Mitomycine C^®^-treated T-cell-depleted splenocytes loaded with the indicated peptide concentrations. ^3^H-thymidine was added for the last 12 h of culture. Error bars represent 1 SD. Data from one experiment representative of three experiments performed in triplicate wells. **(B)** CD4^+^ T cells induced either with WTGAD65 peptide (left panels) or CCGAD65 peptide (right panels) were analyzed by FACS at day 10 of stimulation. Results are from one experiment representative of three independent experiments. Additional information can be found as Figures S3 and S4 in Supplementary Material.

To establish that cCD4^+^ T cells were elicited by peptide immunization, CD4^+^ T splenocytes were isolated from CCGAD65-immunized female NOD mice and expanded *in vitro* in the presence of CCGAD65-loaded APCs. Cells proliferated in the presence of either CCGAD65 peptide or the corresponding WTGAD65 peptide (Figure 2A). Of note, WTGAD65 peptide elicits a stronger response in CD4^+^ T cells. Indeed, although the antigenic stimulation is stronger with the CCGAD65 peptide, this leads to TCR degradation, which can be responsible for a weaker proliferation.

The phenotype at resting state, 14 days after last stimulation with CCGAD65-loaded APCs, was CD3^+^CD4^+^CD8^−^CD25^+^CD44^+^CD62L^−^CD127^−^CD27^−^CD28^−^, indicative of an effector memory phenotype. Some cells were positive for T-Bet and/or GATA-3, but all were negative for Foxp3. The production of cytokines was essentially Il-4 and low IFN-γ. This distinct phenotype was conserved even when cells were repeatedly stimulated *in vitro* with the WTGAD65 peptide, suggesting a terminal differentiation. By contrast, the phenotype expressed by CD4^+^ T cells generated in a similar manner from mice immunized with the WTGAD65 peptide was CD3^+^CD4^+^CD8^−^CD25^+^CD44^+^CD62L^−^CD127^high^CD27^high^CD28^−^ (Figure 2B; Figures S3 and S4 in Supplementary Material). Altogether, this indicates that cells generated with a thioreductase-containing peptide are terminally differentiated effector T cells with a memory phenotype.

**Figure 3:**
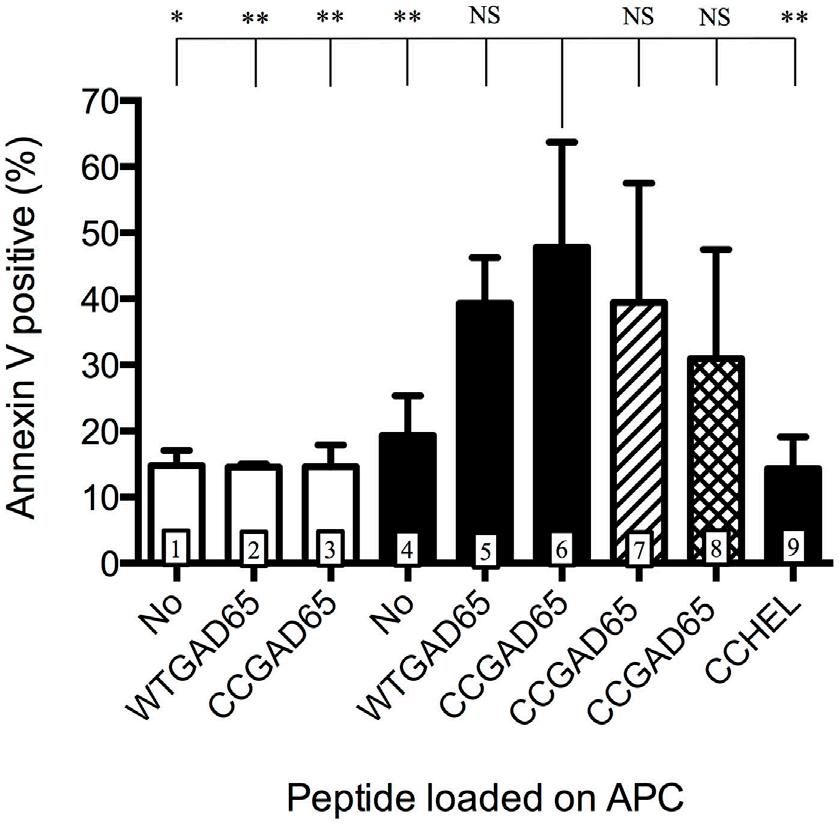
**Antigen-presenting cells are induced into apoptosis by cCD4^+^ T cells**. Splenic B cells isolated by magnetic beads from naive female NOD mice and activated overnight with LPS were cocultured for 24 h with CD4^+^ T cells generated either with the natural GAD65 peptide (white histograms) or with the CCGAD65 peptide (all other columns) in the presence of the indicated peptide (2 μM). Vertical axis represents B cell apoptosis measured by Annexin V binding. Addition of antibody toward FasL is shown in hatched column 7, and inhibitor toward GZB is shown in cross-hatched column 8. Statistical significance was calculated with one-way ANOVA test, *P* < 0.0001 and Dunnett’s multiple comparison test as indicated, *P < 0.05. Error bars represent 1 SD. Data are representative of three independent experiments.

We further evaluated whether such CD4^+^ T cells had acquired cytolytic properties allowing them to induce apoptosis in GAD65-presenting APCs. CD4^+^ T cells generated with the CCGAD65 peptide or the natural peptide were cocultured with naive NOD mouse splenic B cells used as APCs to compare their cytolytic capacity (16). When using cells induced with the natural peptide, B cell apoptosis remained low at 15%. However, when using CCGAD65-induced cells, the rate of B cell apoptosis was increased, and this in the presence of either WTGAD65 (39%) or CCGAD65 peptide (48%). This indicates that CCGAD65-induced cells are able to induce apoptosis in APCs presenting not only the CCGAD65 peptide but also the natural GAD65 peptide as could be encountered *in vivo*. B cell apoptosis was not increased in the absence of peptide (19%) nor when a non-cognate CCHEL peptide was added (14%), confirming that this process remains antigen specific (Figure 3).

Apoptosis can be induced by either the Fas–FasL pathway (7, 17, 18) or by secretion of cytotoxic granules containing granzymes (19). Addition of an antibody toward FasL showed limited but consistent effect by reducing apoptosis from 48 to 39% and GZB inhibitors partially restored B cell survival (31% apoptosis), pointing to a dual mechanism, with GZB showing the strongest effect (Figure 3). Assessing the full implication of FasL or GZB remained limited by the toxicity of the inhibitor itself. GZB involvement was further confirmed by qPCR after stimulation with APCs and the CCGAD65 peptide, with a 200-fold increase in GZB gene expression in cells exposed to the CCGAD65 peptide (Figure S5 in Supplementary Material).

The present results are in keeping with data reported elsewhere (7, 8) and confirm that CD4^+^ T cells acquire an effector memory phenotype together with cytolytic properties after exposure to cognate peptide containing a thioreductase motif.

### CD4^+^ T Cells Stimulated with CxxC Peptides Induce Apoptosis in Bystander T Cells

Conversion of naive and polarized CD4^+^ T cells to cCD4^+^ T cells and induction of apoptosis in antigen-presenting cells might not be sufficient to suppress autoimmunity. The question was therefore extended to bystander CD4^+^ T cells. In view of this description, bystander CD4^+^ T cells are considered as cells activated during the same pathological process. These cells are specific of either the same (auto) antigen, the same epitope, an alternative antigen or an alternative epitope. The underlying goal was to determine whether it was possible to suppress a polyclonal CD4^+^ T cell response using a single epitope from a single antigen.

**Figure 4:**
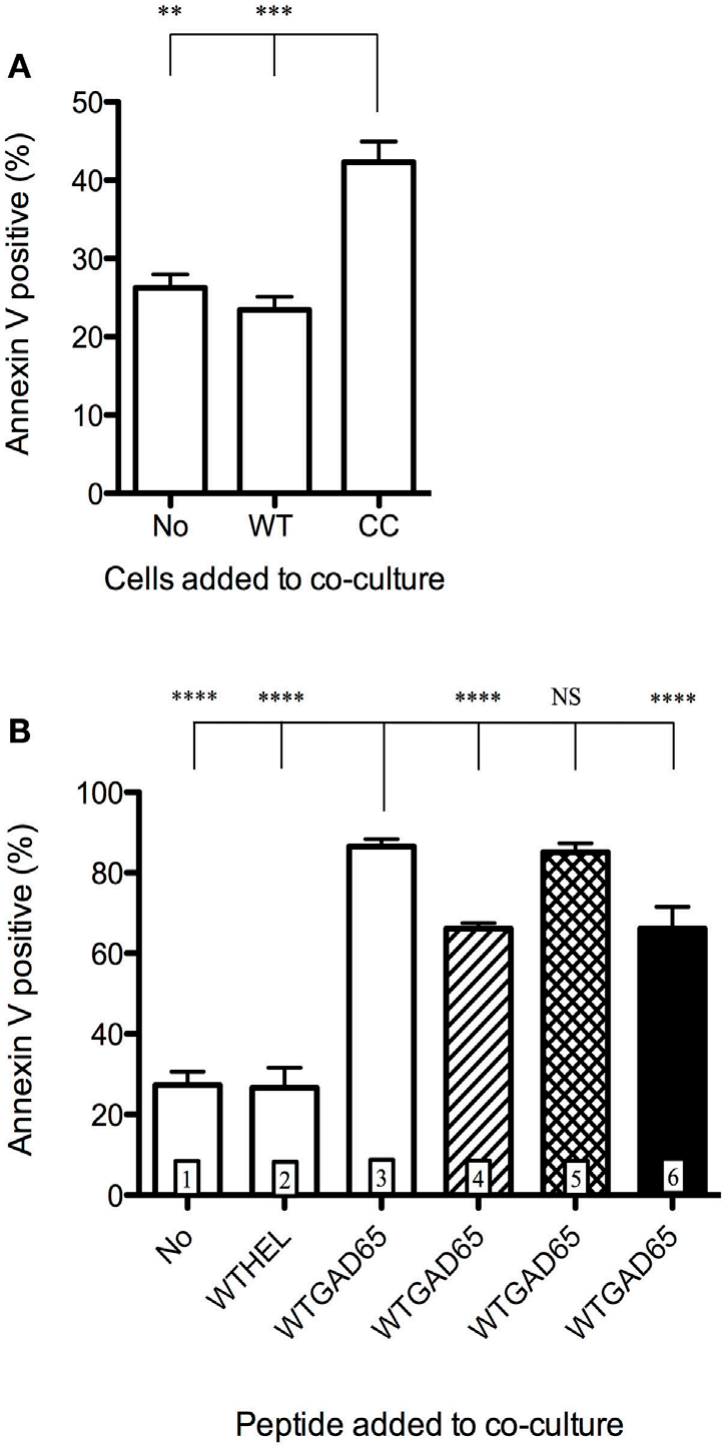
**Induction of apoptosis of bystander CD4^+^ T cells**. CD4^+^ T cells isolated from female NOD mice previously immunized with WTGAD65 peptide and expanded *in vitro* served as bystander CD4^+^ T cells. **(A)** These cells were CFSE stained and cocultured with APCs loaded with WTGAD65 peptide (left column) showing basal mortality rate measured by Annexin V binding (vertical axis). cCD4^+^ T cells generated with CCGAD65 peptide were added to this coculture (right column). A control well was added in which the cCD4^+^ T cells were replaced with the same number of unlabeled CD4^+^ T cells generated with WTGAD65 (middle column). Error bars represent 1 SD. Statistical significance was calculated with one-way ANOVA test, *P* < 0.0001 followed by Dunnett’s multiple comparison test as indicated, *P < 0.05. **(B)** Same CFSE-stained cell line was used in this setting in coculture with APCs and cCD4^+^ T cells generated with CCGAD65 in the presence of the indicated peptide and mortality was measured by Annexin V expression (vertical axis). Hatched column 4 shows the effect of FasL antibody addition to the coculture, cross-hatched column 5 shows addition of GZB inhibitor, and black column 6 shows combination of both blockers. Error bars represent 1 SD. ****P < 0.0001 (One-way ANOVA test and Dunnett’s multiple comparison test). Data are representative of three independent experiments.

To demonstrate that CCGAD65-generated cCD4^+^ T cells were able to induce apoptosis in bystander T cells, these cCD4^+^ T cells were cocultured with APCs and GAD65-specific effector CD4^+^ T cells, in the presence of WTGAD65 peptide, and showed that 42% of effector cells were induced into apoptosis. This was not observed when cCD4^+^ T cells were replaced by effector CD4^+^ T cells (23% mortality) nor when effector cells were used alone without peptide or without cCD4^+^ T cells (26% mortality), excluding effector cell death by lack of stimulation (Figure 4A). Interestingly, cell division in target cells is not affected by replacing unlabeled effector cells with cCD4^+^ T cells (Figure S6 in Supplementary Material). Furthermore, apoptosis was not induced when WTGAD65 peptide was omitted (27% mortality measured by Annexin V) or replaced by a non-cognate CCHEL peptide (27% mortality) as opposed to increased apoptosis when the cognate peptide is used (87% mortality). This indicates that cCD4^+^ T cells killed activated, but not resting bystander CD4^+^ T cells and that killing only occurred in a specific setting. Apoptosis is significantly reduced when antibody to FasL is added during the entire course of the coculture (66% mortality), but not when GZB inhibitor is added (85% mortality). No cumulative effect is seen when antibody to FasL and GZB inhibitor are combined (66% mortality) (Figure 4B).

Apoptosis of bystander CD4^+^ T cells was also seen when using effector CD4^+^ T cells specific for another epitope (GAD65_532–543_). When culturing CFSE-labeled GAD65_532–543_ specific effector cells together with our described cCD4 cells and APCs loaded with both epitopes, we observed bystander T cell apoptosis (74.67%) with Annexin V staining. Importantly, apoptosis was not induced when GAD65_532–543_ was omitted from the coculture.

Our data show that cCD4^+^ T cells elicited by exposure to peptides containing a thioreductase motif acquired the capacity to induce apoptosis of activated bystander T cells and that this activity involved Fas–FasL interaction.

### Transfer of cCD4^+^ T Cells from CCGAD65-Treated Mice Prevents Diabetes and Insulitis in NOD Mice

**Figure 5:**
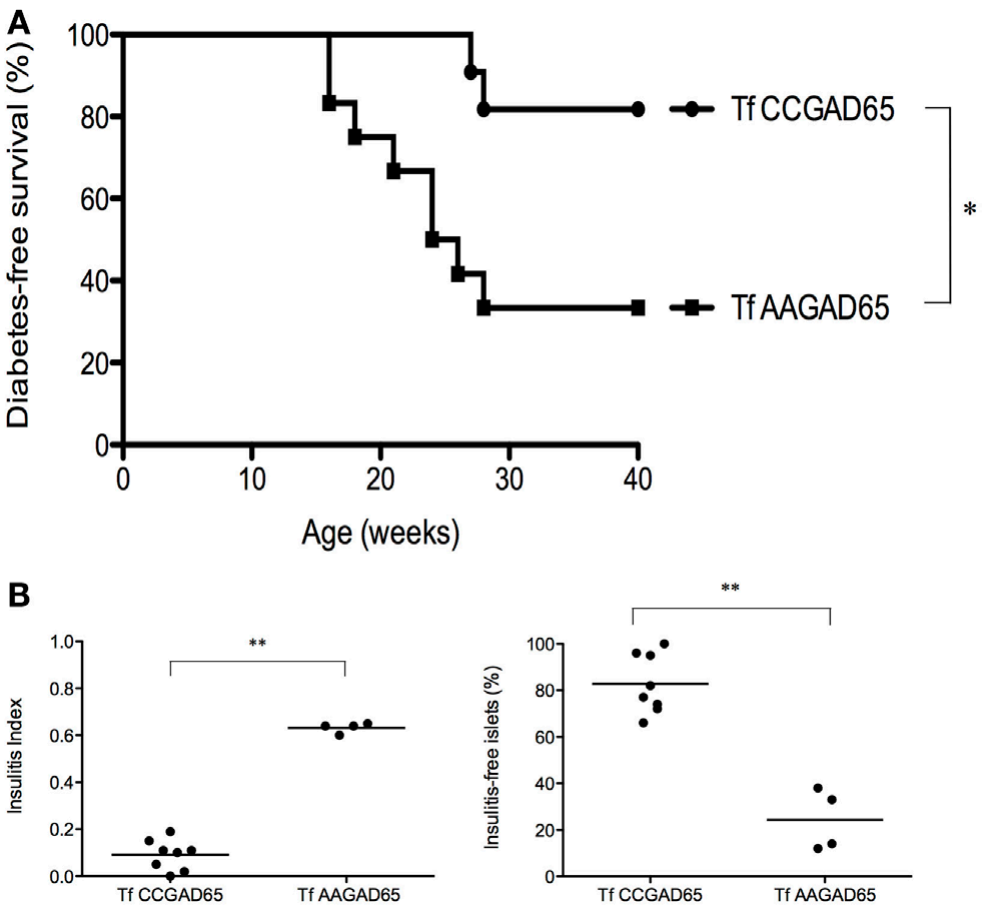
**NOD mice transferred with cCD4^+^ T cells induced by CCGAD65**. Naive mice were passively transferred at 6 weeks of age with cCD4^+^ T cells (2 × 10^5^) obtained from mice preimmunized with CCGAD65 peptide (from 8 to 12 weeks and sacrificed at 15 weeks) and expanded *in vitro* with CCGAD65. Mice were followed up with weekly blood glucose measurements until 40 weeks of age. **(A)** Diabetes-free survival curves are shown for mice transferred with CCGAD65-generated CD4^+^ T cells (*n* = 11), compared to the group transferred with AAGAD65-generated CD4^+^ T cells (*n* = 12) as indicated, *P < 0.05 (Mantel–Cox log-rank test). **(B)** Pancreatic sections of normoglycemic mice at 40 weeks of age transferred either with CCGAD65-induced CD4^+^ T cells or AAGAD65-induced CD4^+^ T cells were stained with H&E and insulitis was scored for at least 40 islets per mouse. Left panel: vertical axis represents insulitis index for each mouse, line indicates mean per group, treated group (*n* = 8) was compared to control group (*n* = 4). Statistical significance was calculated using two-tailed Mann–Whitney, **P < 0.01. Right panel: vertical axis represents percentage of insulitis-free islets per mouse, line indicates mean per group. Treated group was compared to control, **P < 0.01 (two-tailed Mann–Whitney test).

Whether prevention of diabetes was attributable to GAD65-specific cCD4^+^ T cells was evaluated in an adoptive transfer experiment. Figure 5A shows that the diabetes-free survival rate was significantly higher for mice (82%, *n* = 11) transferred at the age of 6 weeks with CD4^+^ T cells isolated from female NOD mice preimmunized with CCGAD65 peptide and expanded *in vitro*, as compared to control mice (33%, *n* = 12) receiving cells generated from mice immunized with the loss-of-function peptide. Of note, cells were phenotyped directly prior to transfer to exclude expansion of unwanted cell lines, including CD8^+^ T cells.

This confirms that GAD65-specific cCD4^+^ T cells are indeed accountable for the observed clinical benefit. In addition, it shows that CD4^+^ T cells generated with the loss-of-function peptide do not elicit any protection against the disease. Mice receiving GAD65-specific cCD4^+^ T cells demonstrated both a drastic reduction in insulitis as mean insulitis index for treated group (*n* = 8) was 0.09 ± 0.02 (SEM) and 0.63 ± 0.01 (SEM) for the control group (*n* = 4) and a clear preservation of insulitis-free islets. Indeed, treated group had 83 ± 4% (SEM) insulitis-free islets as compared to the control group with 24 ± 7% (SEM) (Figure 5B).

### Immunization with CCGAD65 Peptide Prevents Diabetes in *Ins2*^−/−^ NOD Mice

*Ins2*^−/−^ NOD mice develop accelerated diabetes with an onset spanned from 9 to 15 weeks of age in female mice. Elevated incidence of diabetes onset in this model is hypothesized to be related to altered recognition of *proinsulin* and enlarged T cell repertoire specific of insulin in the periphery. Evidence supporting this is the striking difference in anti-insulin autoantibodies with higher prevalence in *Ins2*^−/−^ NOD mice as compared to *Ins2*^+^*^*/*^*^+^ NOD mice (20).

**Figure 6:**
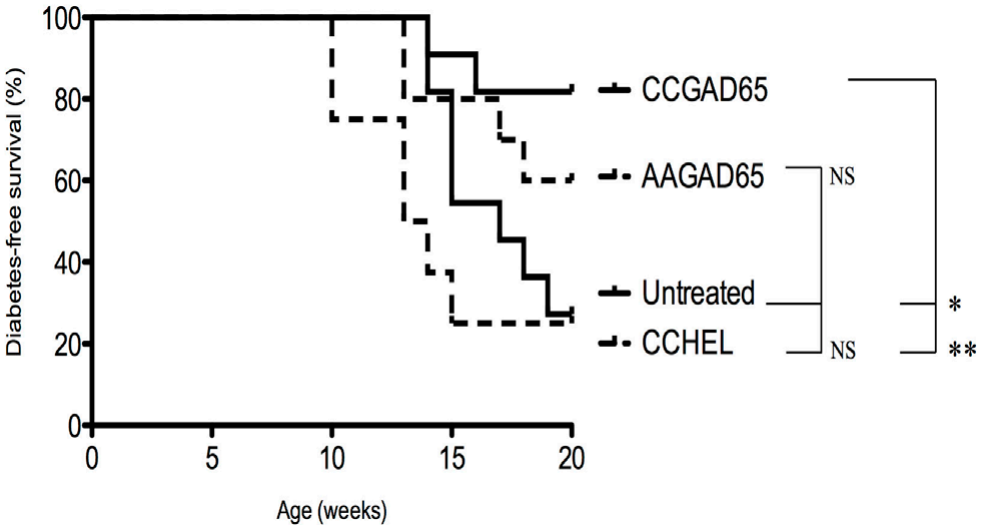
**Diabetes-free survival in female *Ins2^−/−^* NOD mice immunized with CCGAD65 peptide**. In conventional animal facilities, 4-week-old female *Ins2*^−/−^ NOD mice received four weekly subcutaneous injections of 50 μg of CCGAD65 (*n* = 11) or AAGAD65 (*n* = 10) or CCHEL (*n* = 8) peptide in alum as indicated. Control group (*n* = 11) remained untreated. Blood glucose levels were monitored weekly up to 20 weeks of age. Vertical axis represents percentage of diabetes-free survival. Results from two independent experiments, *P < 0.05, **P < 0.005 (Mantel–Cox log-rank test).

Four-week-old female *Ins2*^−/−^ NOD mice were immunized with the indicated peptide, and glycemia was followed on a weekly basis until the age of 20 weeks. Diabetes-free survival rate was increased for CCGAD65-immunized mice (82%) versus AAGAD65-immunized mice (60%), CCHEL-immunized mice (25%), and untreated controls (27%) (Figure 6). Of note, immunization with the non-relevant CCHEL peptide conferred no effect. Prevention of diabetes in this model is further supportive of the bystander effect, though this is not formally demonstrated.

## Discussion

Type 1 diabetes is associated with substantial morbidity and mortality despite replacement therapy with exogenous insulin. Current therapies are neither curative nor antigen specific (21). Interfering with the autoimmune response at the level of effector CD4^+^ T cell activation would fulfill these two conditions by providing early and specific intervention. Tregs are known for their suppressive and regulatory effects on effector cells and are therefore being intensively investigated for their potential to control autoimmune diseases (22, 23). Currently, however, their use is limited by the low frequency of antigen-specific cells and the difficulty to expand them both *in vitro* and *in vivo*.

An alternative and distinct subset of CD4^+^ T cells exerting cytolytic activity has been occasionally described over the last 30 years, mostly in the setting of viral infection (24, 25). cCD4^+^ T cells could potentially be of use in controlling various diseases in which CD4^+^ T cells play a central role if conditions under which they can be generated were better understood. Our work over recent years has succeeded in identifying such conditions, based on inclusion of a thioreductase motif within flanking residues of class II-restricted T cell epitopes (7). This motif, which is characteristic of dicysteinylated glutaredoxins (26), reduces the second domain of the CD4 molecule impacting adaptors of the immune synapse (27). This results in skewing the function and phenotype of the CD4^+^ T cells, which acquire apoptosis-induction activities on APCs presenting the cognate native epitope, as well as on bystander T cells that recognize alternative epitopes on the same APC. The net result of the double action, and the requirement for physical synapse between the cCD4^+^ T cells and the APC presenting the corresponding epitope, is suppression of the response toward the antigen from which the epitope has been derived (intramolecular epitope spreading) and presumably ongoing response toward alternative pathology-associated antigens (intermolecular epitope spreading) (28).

The advantage of designing an epitope where the thioreductase motif is included within the flanking residues lies in the propensity to leave the interface between MHC and TCR unchanged, thus ensuring maintenance of critical interactions (29–31) while gaining the capacity of modifying the nature of the response. This methodology contrasts with that of altered peptide ligands in which amino acid residues from within the MHC class II binding and TCR recognition sequence are mutated (6, 32). Indeed, we have shown that immunization with synthetic peptides encompassing class II-restricted epitopes and a thioreductase motif is efficient to elicit a population of cCD4^+^ T cells in various settings, including models of experimental asthma and of EAE (data not shown).

We show here that a class II-restricted epitope of GAD65, a key autoantigen involved in type 1 diabetes pathogenesis (9, 10), administered by direct immunization to female NOD mice inhibits the development of diabetes (83% in untreated mice versus 57% in CCGAD65-treated mice) and reduces insulitis in association with induction of antigen-specific cCD4^+^ T cells. We further show that cCD4^+^ T cells constitute the likely active ingredient of such effects from passive transfer experiments.

Reduction of the severity of insulitis is the most striking finding, which is strictly dependent on the presence of a pathology-associated epitope and of the presence of a thioreductase motif. In our cohort, as in others (33), insulitis is present in virtually 100% of female NOD mice. Strikingly, up to 22% of CCGAD65-treated mice showed no evidence of insulitis and about 20% showed a mild degree of insulitis. These findings suggest that such mice preserve a full capacity of producing insulin even after prolonged observation (40 weeks).

The reasons as to why not all treated mice respond to therapy with a similar benefit are multiple. Immunization is initiated at week 4 and requires 4 weeks, whereas infiltration of islets with autoreactive CD4^+^ T cells is apparent in between 4 and 6 weeks of age (34). The model itself is heterogeneous, depending on a large number of factors, such as the nature of the microbiotoma (35), the degree of insulin sensitivity (36), and, possibly, the stochastic nature of the first triggering event (37). Of note, we carried out our experiments with GAD65, which is not expressed by beta cells unless under stress conditions.

Interestingly, mice treated with a loss-of-function epitope show a tendency toward increased frequency in diabetes-free animals, while showing no difference on insulitis as compared to control mice, and show no capacity to elicit CD4^+^ T cells with cytolytic capacity. Transfer experiments with cells elicited with loss-of-function epitope likewise show no difference in terms of frequency of diabetes-free animals at 40 weeks. These data point to a possible impact of loss-of-function epitope, which would match published results (38), but which is clearly distinct from the effects observed with peptides used in the present approach. One possibility remains that the pool of effector CD4^+^ T cells is reduced by overstimulation and activation-induced cell death (AICD) resulting from recurrent injections of an epitope in natural sequence (39), or that such peptides elicit Tregs (40).

The data reported here indicate that Fas–FasL interactions play a role in induction of apoptosis of activated bystander CD4^+^ T cells, while GZB is prominent for apoptosis of APC. Interestingly, apoptosis of bystander CD4^+^ T cells occurs only when such cells are activated on the same APC, illustrating the requirement for cell–cell contact and independence from cytokine production. As in Tregs, cCD4 T cells are resistant to apoptosis by mechanisms that are not yet fully understood. Strikingly, these cCD4^+^ T cells acquire a stable and terminal phenotype characteristic of differentiated effector memory T cells.

Another parameter that was of concern for this study is the possible impact of the maintenance of animals in SPF facilities. It is known that the incidence of diabetes in the NOD mice is greatly enhanced by keeping mice under SPF conditions and many reasons have been raised to explain this (15). However, much less information exists on the effect of SPF conditions on insulitis. Our observations (unpublished), in keeping with others, indicate that a more aggressive form of insulitis occurs under such conditions, due to, *inter alia*, increased number of NK cells and reduced number of Tregs. It was therefore of importance to demonstrate that our approach was efficient under free and SPF conditions.

CD4^+^ T cells might, however, not be the only T cell subset relevant to type 1 diabetes pathogenesis. A pathogenic role for CD8^+^ T cells in autoimmune diabetes has indeed been described (41, 42), possibly as an initiating event. We, however, suggest that eliminating CD4^+^ T cells using the present methodology could also prevent activation of CD8^+^ T cells.

A popular tenet in autoimmunity is that there could be one primary autoantigen that initiates pathogenesis. Tissue destruction through targeting of this antigen could further release other ones, thus amplifying the autoimmune cascade through a phenomenon known as epitope spreading. Epitope spreading occurs once the disease process is triggered, and there is no doubt that several β-cell antigens, and not only GAD65, are involved in type 1 diabetes (6, 43). The present technology using a single epitope of a single β-cell antigen potentially prevents responses toward alternative epitopes and perhaps even alternative antigens, provided the latter are presented by the same APC. This condition is easily achieved considering that the main site at which the cCD4^+^ T cells control the immune response is in the lymph nodes draining the diseased organ, a location at which much of the autoantigens released into the affected organs are processed for presentation to the immune system. As type 1 diabetes is a chronic progressive autoimmune disease evolving with an increasing number of autoantigens over time, the ability to impact multiple epitopes would represent a great asset. Efficacy in controlling diabetes onset in the *Ins2*^−/−^ NOD mouse model defined by a T cell repertoire directed essentially toward insulin by immunization with a thioreductase-containing GAD65 peptide further anticipates a bystander effect toward alternative antigens.

Although acknowledging the limitations of the NOD mouse strain to predict effects in humans, this spontaneous model remains, in our view, the best available preclinical whole animal system representative of human type 1 diabetes (44), namely, allowing us to examine long-term consequences of a therapy. Noticeably, while a short course of immunization was sufficient to sustain long-standing efficacy in a context of continuing exposure to islet cell antigens, no adverse effects on mouse general status were observed over the 40 weeks follow-up period.

## Conclusion

Altogether, preclinical efficacy has been established in the NOD mouse model by assessing glycemia and insulitis after active immunization with CCGAD65 peptide or transfer of cCD4^+^ T cells induced with CCGAD65. Additionally, these cCD4^+^ T cells have been shown to induce apoptosis of the APC presenting the epitope and of bystander T cells. Consequently, the present approach offers a promising antigen-specific therapy for type 1 diabetes patients without compromising their general immune status.

## Author Contributions

JMSR elaborated the initial concept; EMA, LVE, VC, and JMSR contributed to the design of the studies; EMA and LVE carried out animal experiments; EMA and VC performed cell cultures and *in vitro* experiments; EMA, LVE, and VC analyzed the results; EMA and JMSR wrote the manuscript; and JMSR is the guarantor of this work.

## Conflict of Interest Statement

The authors declare that the research was conducted in the absence of any commercial or financial relationships that could be construed as a potential conflict of interest.
