# Corrigendum: Thioreductase-Containing Epitopes Inhibit the Development of Type 1 Diabetes in the NOD Mouse Model

**DOI:** 10.3389/fimmu.2018.01600

**Published:** 2018-07-09

**Authors:** Elin Malek Abrahimians, Luc Vander Elst, Vincent A. Carlier, Jean-Marie Saint-Remy

**Affiliations:** ^1^Center for Molecular and Vascular Biology, University of Leuven, Leuven, Belgium; ^2^ImCyse SA, Leuven, Belgium

**Keywords:** type 1 diabetes, NOD mouse, cytolytic CD4^+^ T cells, antigen-specific, MHC class II epitopes

In the original article, there was an error in the wording concerning the production of cytokines.

A correction has been made to the section Results, Subsection CCGAD65 Peptide Elicits CD4+ T Cells with a Unique Phenotype and Cytolytic Properties, Paragraph 2:

The phenotype at resting state, 14 days after last stimulation with CCGAD65-loaded APCs, was CD3^+^CD4^+^CD8^−^CD25^+^CD44^+^CD62L^−^CD127^−^CD27^−^CD28^−^, indicative of an effector memory phenotype. Some cells were positive for T-Bet and/or GATA-3, but all were negative for Foxp3. The production of cytokines was essentially Il-4 and low IFN-γ. This distinct phenotype was conserved even when cells were repeatedly stimulated *in vitro* with the WTGAD65 peptide, suggesting a terminal differentiation. By contrast, the phenotype expressed by CD4^+^ T cells generated in a similar manner from mice immunized with the WTGAD65 peptide was CD3^+^CD4^+^CD8^−^CD25^+^CD44^+^CD62L^−^CD127^high^CD27^high^CD28^−^ (Figure 2B; Figures S3 and S4 in Supplementary Material). Altogether, this indicates that cells generated with a thioreductase-containing peptide are terminally differentiated effector T cells with a memory phenotype.

The authors apologize for this error and state that this does not change the scientific conclusions of the article in any way.

The original article has been updated.

## Conflict of Interest Statement

The authors declare that the research was conducted in the absence of any commercial or financial relationships that could be construed as a potential conflict of interest.

