# Supplementary material for: Thioreductase-Containing Epitopes Inhibit the Development of Type 1 Diabetes in the NOD Mouse Model
Source: Front Immunol. 2016 Mar 2;7:67. doi: 10.3389/fimmu.2016.00067 (PMC4773585; doi:10.3389/fimmu.2016.00067)
Supplement: Supplementary file 1 [file data_sheet_1.docx]

Supplementary Material

Thioreductase-containing epitopes inhibit the development of type 1 diabetes in the NOD mouse model

Elin Malek Abrahimians^1,2^, Luc P.M. Vander Elst^1,2^, Vincent A. Carlier^1,2^ and Jean-Marie Saint-Remy^1,2*^

^1^Center for Molecular and Vascular Biology, University of Leuven, Leuven, Belgium

^2^ImCyse SA, Leuven, Belgium

*** Correspondence:** Jean-Marie Saint-Remy, ImCyse, Bioincubator II, Gaston Geenslaan 1, Leuven 3001, Belgium, jean-marie.saint-remy@med.kuleuven.be

**Supplementary Figure 1**

**Supplementary Figure 1. NOD mice immunized with CCGAD65 peptide in conventional animal facilities**. **(A)** 4-week-old female NOD mice (n=22) received 4 subcutaneous injections of 50 µg of CCGAD65 peptide in alum with a 7-10 day interval. Control group (n=21) remained untreated. Blood glucose levels were monitored weekly up to 40 weeks of age. Diabetes-free survival rate was significantly increased for immunized (82%) *versus* untreated controls (38%) at 40 weeks of age. Diabetes-free survival curves are shown and vertical axis represents percentage of diabetes-free survival, **P <0.005 (Mantel-Cox log-rank test). Data are representative of two independent experiments. **(B)** Pancreatic sections of normoglycemic mice at 40 weeks of age from either CCGAD65-immunized (n=16) or untreated group (n=5) were stained with H&E and insulitis was scored by examining a minimum of 40 non-overlapping islets per mouse. Normoglycemic CCGAD65-immunized mice showed significantly reduced insulitis index (II) at 40 weeks with a mean II of 0.10 ± 0.03 (SEM) as compared to 0.45 ± 0.06 (SEM) for the untreated group. Notably, the immunized group benefited from a significantly higher number of insulitis-free islets with 75% ± 6 (SEM) intact islets as opposed to 34% ± 7 (SEM) in the untreated group, and 4 mice out of 16 in the treated group showed absolutely no signs of insulitis at 40 weeks of age. (Left panel) Vertical axis represents insulitis index for each mouse and horizontal bar indicates mean per group. Statistical significance was calculated using two-tailed Mann-Whitney, **P <0.005. (Right panel) Vertical axis represents percentage of insulitis-free islets per mouse, line indicates mean per group, **P <0.01 (two-tailed Mann-Whitney test). Data are representative of two independent experiments. **(C)** Representative histopathological images (with 40x magnification) of pancreas sections from either immunized (top panel, showing insulitis-free islet) or non-immunized (bottom panel, showing massive inflammatory infiltrate of the islet) normoglycemic NOD mice at the age of 40 weeks.

**Supplementary Figure 2**

**Supplementary Figure 2.** **Immunization with CCGAD65 peptide prevents insulitis in NOD mice in SPF animal facilities**. Mice were immunized as described in Figure 1. **(A)** Pancreatic sections of mice sacrificed after remaining normoglycemic during 40 weeks (top panels) or after two consecutive weekly glycemias above 300mg/dl (bottom panels) were stained with H&E. Insulitis was scored by examining a minimum of 40 islets per mouse. Individual II (left panels) and percentage of insulitis-free islets (right panels) are represented for each group with line representing mean. In normoglycemic mice, mean insulitis indexes were calculated at, from left to right, 0.47 for mice immunized with CCGAD65 peptide in alum, 0.65 for mice immunized with AAGAD65 peptide in alum, 0.60 for mice receiving CCHEL peptide in alum or alum alone and at 0.71 for untreated mice (P <0.0001, Kruskal-Wallis ANOVA test). Mean insulitis-free percentage in normoglycemic mice was 29.6% for CCGAD65 group, 14.2% for AAGAD65 group, 17.5% for CCHEL group, 17.6% for alum group and 16.5% in untreated group (P <0.05, Kruskal-Wallis ANOVA test). In diabetic mice, mean insulitis indexes were at 0.54 in CCGAD65 group, 0.62 in AAGAD65 group, 0.65 in CCHEL group, 0.68 in alum group and 0.69 in untreated group (P <0.0005, Kruskal-Wallis ANOVA test). Mean insulitis-free percentage in hyperglycemic mice was 16.9% for CCGAD65 group, 8.8% for AAGAD65 group, 7.7% for CCHEL group, 4.1% for alum group and 5.3% in untreated group (P <0.01, Kruskal-Wallis ANOVA test). **(B)** Pancreatic sections of some normoglycemic mice were analyzed at 20 weeks of age and showed similar results. Mean insulitis indexes were of 0.52 in CCGAD65 group, 0.69 in AAGAD65 group, 0.72 in CCHEL group, 0.76 in alum group and 0.63 in untreated group, P <0.05, Kruskal-Wallis ANOVA test).

**Supplementary Figure 3**

**Supplementary Figure 3. Characterization of WT versus CCGAD65-induced CD4^+^ T cells.** Cells obtained from mice immunized either with the WTGAD65 peptide (black histograms) or the CCGAD65 peptide (gray histograms) were stained 10 days after last stimulation for the indicated surface markers. 99% ± 0.9 (SD) of WTGAD65 generated cells are positive for CD44, 59.2% ± 6.0 (SD) for CD27 and 60.6% ± 12.1 (SD) for CD127, while 99.1% ± 1.0 (SD) of CCGAD65 generated cells are positive for CD44, 0.7% ± 0.6 (SD) for CD27 and 2.0% ± 1.9 (SD) for CD127. Results are show as mean percentages of three independent experiments. Error bars represent 1 SD.

**Supplementary Figure 4**

**Supplementary Figure 4. Characterization of CCGAD65-induced CD4^+^ T cells. (A)** Representative FACS plots of CD25, CD8, CD28 and CD62L staining of CD4^+^ T cells induced either with WTGAD65 peptide (left panels) or CCGAD65 peptide (right panels) at day 10 of stimulation. **(B)** At 24h after stimulation, cells were stained for intracellular Foxp3 and analyzed by flow cytometry. Overlay of Foxp3 expression is shown for CD4^+^ T cells induced either with WTGAD65 peptide (left panel) or CCGAD65 peptide (right panel) in comparison with isotype control as indicated. Results are from one experiment representative of three independent experiments.

**Supplementary Figure 5**

**Supplementary Figure 5.** **CD4^+^ T cells generated with CCGAD65 peptide express higher levels of GZB, FasL and CD107a. (A)** GZB gene expression was analyzed by qPCR in CD4^+^ T cells induced with the natural GAD65 peptide (WTGAD65) or with the CCGAD65 peptide 24 hours after stimulation with APCs-loaded with the respective peptide. Vertical axis represents fold change in gene expression. **(B)** Overlay of FasL expression is shown in same setting comparing CD4^+^ T cells induced either with WTGAD65 peptide or CCGAD65 peptide and isotype control as indicated. **(C)** Representative FACS plots of CD107a of CD4^+^ T cells induced either with WTGAD65 peptide (left panel) or CCGAD65 peptide (right panel) at day 10 of stimulation. Results are from one experiment representative of three independent experiments.

**Supplementary Figure 6**

**Supplementary Figure 6.** **Cell division in bystander CD4^+^ T cells was evaluated by CFSE dilution assay.** CFSE staining showed that non-stimulated effector CD4^+^ T cells show no cell division (1.2%, top panel), while effector CD4^+^ T cells stimulated with APCs presenting cognate peptide in the presence of cCD4^+^ T cells express similar cell division pattern (26%, middle panel) as when cCD4^+^ T cells are replaced with unlabeled effector CD4^+^ T cells (25.8%, bottom panel).
